# Bioinformatic analysis of cis-regulatory interactions between progesterone and estrogen receptors in breast cancer

**DOI:** 10.7717/peerj.654

**Published:** 2014-11-18

**Authors:** Matloob Khushi, Christine L. Clarke, J. Dinny Graham

**Affiliations:** Centre for Cancer Research, Westmead Millennium Institute, Sydney Medical School—Westmead, University of Sydney, Australia

**Keywords:** Transcription factors, Estrogen receptor alpha, Progesterone receptor, ERα, ESR1, PR, Breast cancer, T47D, BiSA, Genomic region database

## Abstract

Chromatin factors interact with each other in a cell and sequence-specific manner in order to regulate transcription and a wealth of publically available datasets exists describing the genomic locations of these interactions. Our recently published BiSA (Binding Sites Analyser) database contains transcription factor binding locations and epigenetic modifications collected from published studies and provides tools to analyse stored and imported data. Using BiSA we investigated the overlapping cis-regulatory role of estrogen receptor alpha (ER*α*) and progesterone receptor (PR) in the T-47D breast cancer cell line. We found that ER*α* binding sites overlap with a subset of PR binding sites. To investigate further, we re-analysed raw data to remove any biases introduced by the use of distinct tools in the original publications. We identified 22,152 PR and 18,560 ER*α* binding sites (<5% false discovery rate) with 4,358 overlapping regions among the two datasets. BiSA statistical analysis revealed a non-significant overall overlap correlation between the two factors, suggesting that ER*α* and PR are not partner factors and do not require each other for binding to occur. However, Monte Carlo simulation by Binary Interval Search (BITS), Relevant Distance, Absolute Distance, Jaccard and Projection tests by Genometricorr revealed a statistically significant spatial correlation of binding regions on chromosome between the two factors. Motif analysis revealed that the shared binding regions were enriched with binding motifs for ER*α*, PR and a number of other transcription and pioneer factors. Some of these factors are known to co-locate with ER*α* and PR binding. Therefore spatially close proximity of ER*α* binding sites with PR binding sites suggests that ER*α* and PR, in general function independently at the molecular level, but that their activities converge on a specific subset of transcriptional targets.

## Introduction

The ovarian steroid hormones progesterone and estrogen play critical roles in the development and progression of breast cancer and endometriosis (D’Abreo & Hindenburg, 2013; Salehnia & Zavareh, 2013; Shao et al., 2014). These hormones exert their functions by activating specific nuclear receptors, estrogen binds to estrogen receptor (ER*α*) and progesterone binds to progesterone receptor (PR) (Tsai & O’Malley, 1994).

Once activated these receptors bind to their DNA response elements and regulate transcription of target genes. ER*α* and PR, along with human epidermal growth factor receptor 2 (HER2), are used to classify phenotypes in breast cancers and to predict response to specific therapies (Cadoo, Fornier & Morris, 2013; Kittler et al., 2013). A high number of ER*α* positive breast cancers are also PR positive (Cadoo, Fornier & Morris, 2013; Penault-Llorca & Viale, 2012). Furthermore, studies from animal models and clinical trials have shown that progesterone via its receptor PR is a major player in development and growth of breast cancer and uterine fibroids, however, PR inhibits the development of estrogen-driven endometrial cancer (Ishikawa et al., 2010; Kim, Kurita & Bulun, 2013). Many recent reviews highlight the importance of the role that progesterone and estrogen play via their receptors in various types of breast cancers (Abdel-Hafiz & Horwitz, 2014; Kalkman, Barentsz & van Diest, 2014; Obiorah et al., 2014; Wang & Di, 2014; Yadav et al., 2014). Therefore it is important to understand how ER*α* and PR work together in regulating a number of cellular pathways, and clinical and molecular research on these factors continue to unveil new insights (Bulun, 2014).

It is acknowledged that ER*α* and PR binding, as well as that of other steroid hormone receptors, is assisted by binding of the pioneer transcription factor FOXA1 (Ballare et al., 2013; Lam et al., 2013) to condensed chromatin, therefore, the interactions of FOXA1 with other factors have been well studied (Augello, Hickey & Knudsen, 2011; Bernardo & Keri, 2012). There are a number of publications that have studied PR binding sites in progesterone-treated breast and other tissues (Ballare et al., 2013; Clarke & Graham, 2012; Yin et al., 2012). Many studies have also published ER*α* binding sites (Joseph et al., 2010; Schmidt et al., 2010; Tsai et al., 2010). However there is lack of investigation into the combined action of the two factors on DNA. Therefore in this report we investigated the interaction of these nuclear receptors on DNA. Our previously published BiSA database (Khushi et al., 2014) contains a number of datasets describing ER*α* and PR binding sites for various cell lines, therefore, we investigated the binding pattern of these factors in the T-47D breast cancer cell line. T-47D cells are derived from metastatic female human breast cancer and are known to be ER*α* and PR positive and their growth is simulated by the treatment of estrogen (Chalbos et al., 1982; Ström et al., 2004).

## Methods

PR data were taken from the study of Clarke & Graham (2012) and ER*α* data were obtained from the ENCODE project (Gertz et al., 2012). PR data were obtained by treating T47D cells with the progestin ORG2058 for 45 min, followed by PR-specific chromatin immunoprecipitation and deep sequencing (ChIP-Seq). Gertz et al. studied ER*α* binding sites by treating with estradiol (E2), GEN (Genistein) and BPA (Bisphenol A) and conclude that compared to E2, GEN and BPA treatment results in fewer ER*α* binding sites and less change in gene expression. We selected the E2-treated dataset for our study. Datasets from both studies were of 36 base pair lengths on the Illumina platform. The PR data were generated using an Illumina Genome Analyzer IIx while ER*α* libraries were sequenced on Illumina HiSeq 2000. The data used in this study have been derived from peer-reviewed publications, suggesting that they are of an acceptable quality, in addition we also ensured standard quality control checks prior to our re-analysis of the raw data. The two studies used different genome assemblies and different tools to align the reads and to call the peaks. Therefore, to remove any biases we re-analysed the raw ER*α* and PR data. We mapped the raw data to the GRCh37/hg19 assembly using Bowtie version 2 (Langmead & Salzberg, 2012). The aligned replicates were merged using Picard tools (Li et al., 2009) and Model-based Analysis of ChIP-seq Algorithm (MACS) version 1.4.2 (Zhang et al., 2008) was employed, with default settings, to identify PR and ER*α* binding regions in the two datasets. Regions associated with greater than 5% false discovery rate (FDR) were removed (Zhang et al., 2008).

We performed motif analysis using HOMER software (Heinz et al., 2010). HOMER employs a differential motif discovery algorithm by comparing two sets of sequences and quantifying consensus motifs that are differentially enriched in a set. HOMER automatically generates an appropriate background sequence matched for the GC content to avoid bias from CpG Islands. The tool is exclusively written for analysing DNA regulatory elements in ChIP-Seq experiments and has been used in number of high impact publications (Berman et al., 2012; Wang et al., 2011b; Xie et al., 2013).

Overlapping features were studied in BiSA (Khushi et al., 2014). BiSA is a bioinformatics database resource that can be run on Windows as a personal resource or web-based under Galaxy (Goecks et al., 2010) as a collaborative tool. BiSA is pre-populated with published transcription factor and histone modification datasets and allows investigators to run a number of overlapping and non-overlapping genomic region analyses using their own datasets, or against the pre-loaded Knowledge Base. Overlapping features can be visualised as a Venn diagram and binding regions of interest can also be annotated with nearby genes. BiSA also provides an easy graphical interface to find the statistical significance of observed overlap between two genomic region datasets by implementing the IntervalStat tool (Chikina & Troyanskaya, 2012). The tool calculates a *p*-value for each peak region by comparing a region from the query dataset to all regions in a reference dataset. The tool restricts the analysis to regions that are within a domain dataset which can be a whole genome or can be possible interval locations such as promoter proximal regions. Based on IntervalStat calculated *p*-values BiSA calculates a summary statistic that we refer to as the Overlap Correlation Value (OCV). The OCV ranges from 0 to 1, the closer the value to 1 the stronger the significance of overlap of two datasets. The OCV represents the fraction of regions in the query dataset with a *p*-value less than a specified threshold. In BiSA, we have set the threshold *p*-value to 0.05 and used a number of domains such as whole genome and promoter proximal regions for this analysis.

We also investigated the spatial correlation of regions of whole datasets being closer to each other by Binary Interval Search (BITS) (Layer et al., 2013) and Genometricorr (Favorov et al., 2012). BITS implements a Monte Carlo simulation by comparing actual overlapping regions to random observed overlap. Genometricorr considers one genomic region set as a reference and other set as a query and provides four asymmetric pair-wise statistical tests (i) relative distance also called local correlation, (ii) absolute distance, (iii) Jaccard statistic and (iv) projection statistical tests. In local correlation the significance of relative distance between the genomic regions is measured by Kolmogorov–Smirnov test, in absolute distance test the significance of base pair distance among the regions is measured by permutation test, Jaccard statistic takes into account the ratio of intersecting bases to the union base pairs. A projection test calculates the overlapping centre points of query to reference regions and finds the significance of result outside of the null expectation by binomial test (Favorov et al., 2012). We performed 10,000 simulations for BITS and Genometricorr statistical tests.

We performed functional annotation of ER*α*-PR common cis-regulatory regions using GREAT (Genomic Regions Enrichment of Annotations Tool) (McLean et al., 2010). GREAT incorporates annotations from 20 ontologies covering gene ontology, phenotype data, human disease pathways, gene expression, regulatory motifs and gene families. We performed GREAT annotation using its default settings. A region was considered to have a proximal association with a gene if it was within 5 kb upstream or 1 kb downstream of the transcription start site (TSS). Regions outside this distance and up to 1,000 kb from the TSS to the next gene proximal region were considered to have a distal association.

## Results

Analysis of PR and ER*α* ChIP-seq data from T-47D breast cancer cells revealed 22,152 PR and 18,560 ER*α* binding regions with FDR <5%. HOMER motif analysis on the top ranked 1,000 regions by peak score revealed the strong presence of a PRE motif (59.40%) and ERE motif (48.80%) (Tables 1 and 2). These were the most statistically significant motifs identified, in agreement with other studies (Kim, Kurita & Bulun, 2013; Lin et al., 2007). In addition, in PR binding regions we found motifs for the transcriptional partners FOXA1 and AP-2 (TFAP2C) as other top ranked motifs. The transcription factor activator protein 2C (TFAP2C) is known to be involved in normal mammary development, differentiation, and oncogenesis (Cyr et al., in press; Lal et al., 2013; Woodfield et al., 2010). Interestingly PR motifs were present in 344 (34.4%) of the 1,000 top ranked ER*α* binding regions. Consensus FOXA1 motifs were also detected in 27% of PR binding regions and 24% of regions bound by ER*α*. FOXA1 is a member of the forkhead family of transcription factors, which are known to bind and reconfigure condensed chromatin to enable the binding of other transcription factors (Bernardo & Keri, 2012) . The presence of high quality (*p*-value <1.00e–05) peaks and known conserved PR and ER*α* recognition sequences confirmed the success of the alignment and peak-calling process.

**Table 1:** Motif analysis of PR regions. Known motif analysis of PR top 1,000 regions using Homer software.

Motif	Name	*P*-value	% of targets sequences with motif
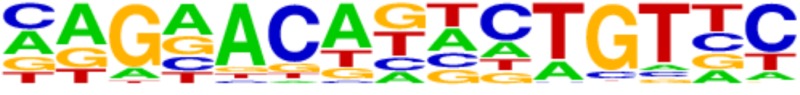	PR(NR)/T47D	1e–123	59.40%
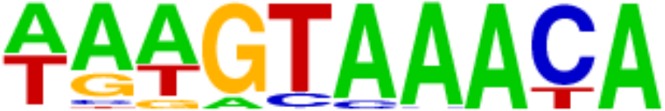	FOXA1(Forkhead)/LNCAP-FOXA1	1e–28	27.10%
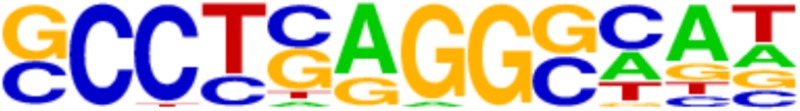	AP-2gamma(AP2)/MCF7-TFAP2C	1e–10	13.70%

**Table 2:** Motif analysis of ER*α* regions. Known motif analysis of ESR1 top 1,000 regions.

Motif	Name	*P*-value	% of targets sequences with motif
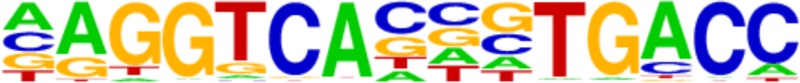	ERE(NR/IR3)/MCF7-ERa	1e–474	48.80%
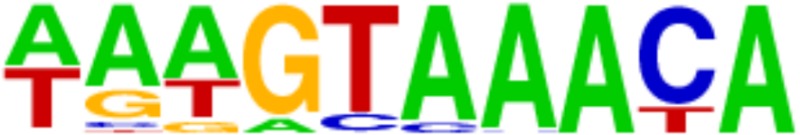	FOXA1(Forkhead)/LNCAP-FOXA1	1e–22	24.30%
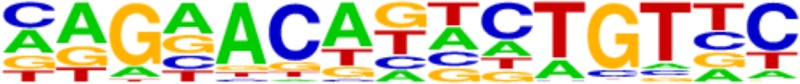	PR(NR)/T47D-PR	1e–20	34.40%

**Figure 1:**
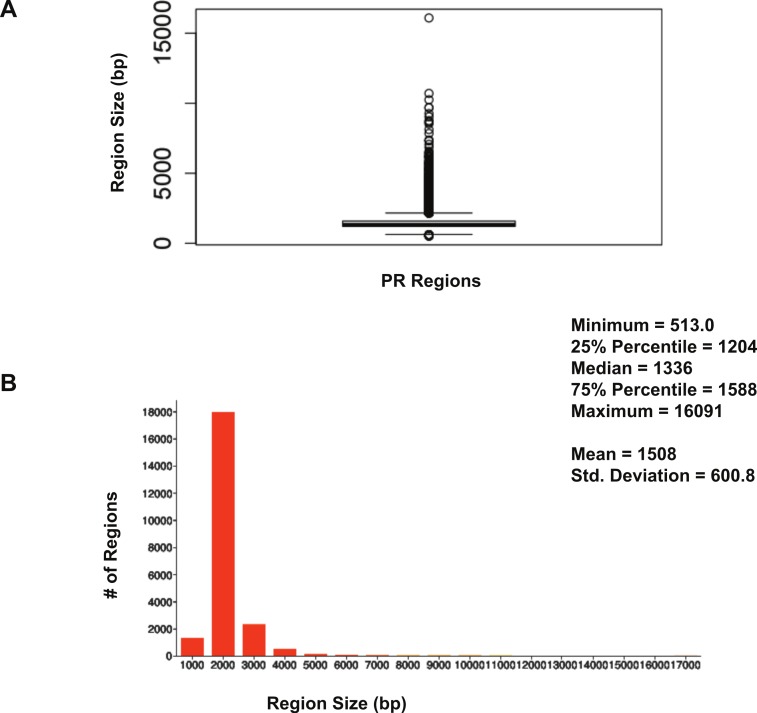
Distribution of PR binding region sizes. (A) Box plot with mean and median information. (B) Histogram of region sizes with bin size 1,000.

**Figure 2:**
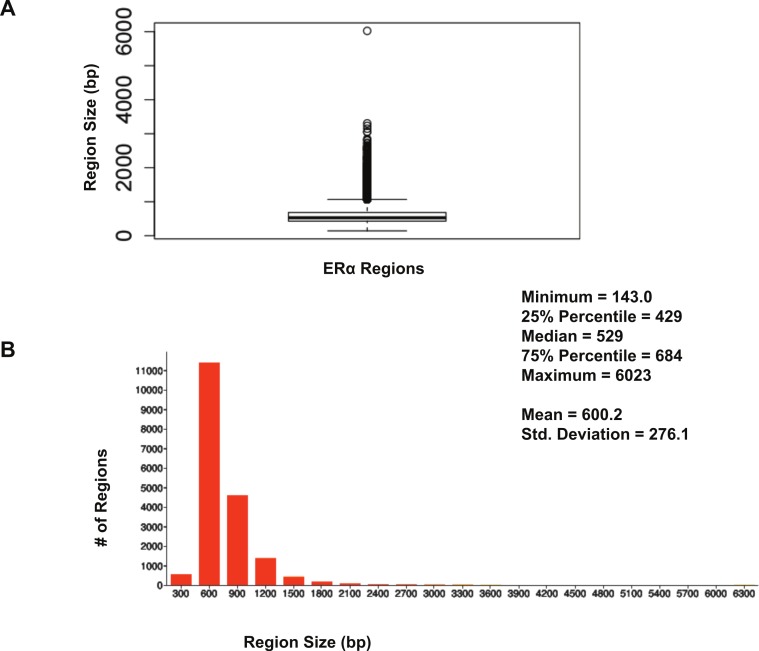
Distribution of ER*α* binding region sizes. (A) Box plot with mean and median information. (B) Histogram of ER*α* region sizes with bin 200.

The size distribution of ER*α* (18,560 regions) and PR (22,152 regions) binding regions were visualised by drawing a histogram and box plot (Figs. 1 and 2). Mean PR binding region size was 1508 with a median of 1336. In contrast, ER*α* binding regions were on average half the size of PR binding regions, with a mean size of 601 and median 529. Most PR binding regions (∼94%) were greater than 1 kb, whereas most ER*α* binding regions (∼95%) were less than 1 kb. The longer PR regions may be due to longer input DNA fragment lengths in the original samples (Kharchenko, Tolstorukov & Park, 2008; Landt et al., 2012).

### Limited overlap of ER*α* and PR regions

**Figure 3:**
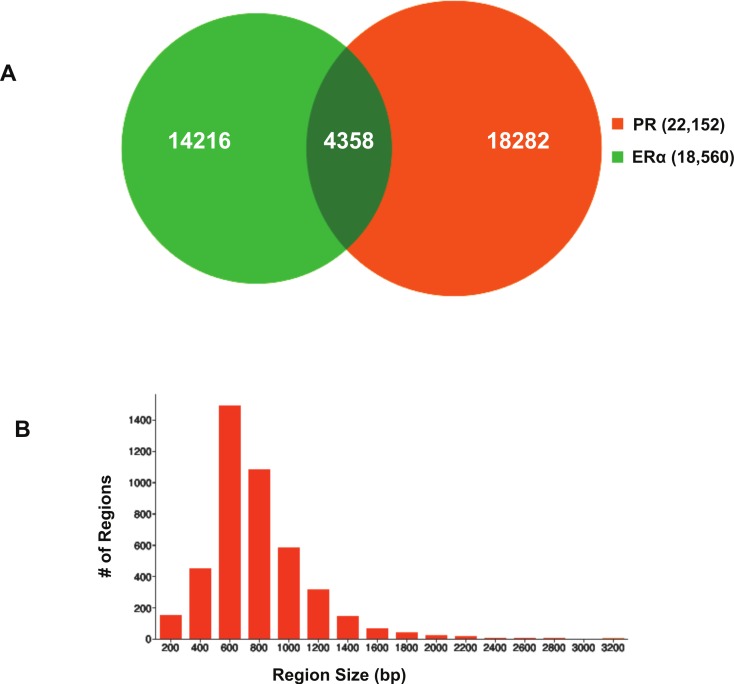
Visualisation of ER*α* and PR binding region overlap. (A) Venn diagram showing overlap between ER*α* and PR data. The 4,344 ER*α* binding regions overlap with 3,870 unique PR binding regions making up 4,358 overlapping sections. (B) Region sizes of 4,358 regions common to the ER*α* and PR datasets.

**Figure 4:**
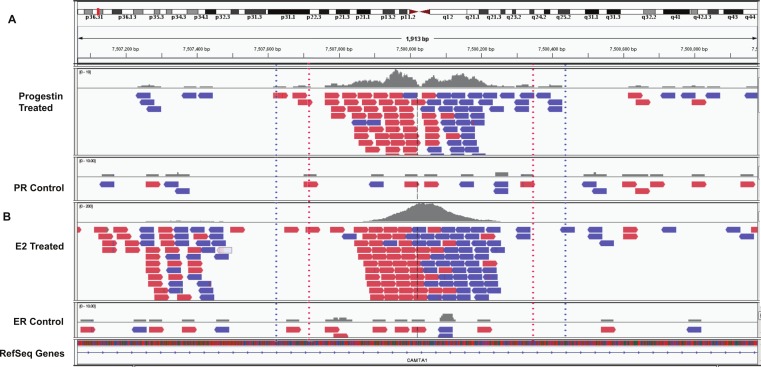
Example overlapping region. IGV snapshot of PR binding region at chr1:7507615–7508428 (marked by blue dotted lines) and ER*α* binding region (marked by red dotted lines). (A) Progestin treated and control samples. (B) Estradiol (E2) treated and control sample. The red boxes are reads that mapped to the forward strand and blue boxes are reads that mapped to the reverse strand of the human genome (build hg19).

Using BiSA, we identified that almost one quarter (23.6%) of ER*α* binding regions (4,344) overlap with 3,870 unique PR binding regions. This revealed that some long PR binding regions spanned more than one ER*α* binding region and the reverse was also true for large ER*α* binding regions. In total, we found 4,358 sections that were common to the two datasets. The Venn diagram in Fig. 3A shows this overlap between the two ligand-activated transcription factors. The 4,358 overlapping sections of the regions common to the two datasets were extracted and plotted for their region lengths (Fig. 3B). Out of 4,358 overlapping sections 4,279 (98.2%) were more than 100 bases long, suggesting a strong binding overlap between the two transcription factor data sets. An example of a shared ER*α* and PR binding region is shown in Fig. 4. The 631 bp ER*α* binding region (red dotted lines) is completely contained within the 813 bp PR binding region (blue dotted lines) and the two regions share the peak centre location (Fig. 4).

### Statistical analysis of ER*α*-PR overlap

To determine whether the overlap between ER*α* and PR binding was statistically significant, statistical analysis was performed in BiSA, BITS and Genometricorr. In BiSA, using a whole genome domain and selecting the ER*α* cistrome as query and PR as reference revealed an overlap correlation value of 0.33. The value decreased to 0.26 when PR was selected as query and ER*α* as reference. This showed that, although a considerable proportion of ER*α* binding regions are also bound by PR, the two receptors do not cooperate for binding at all sites. To determine whether the significance of ER*α*-PR binding overlap was greater in functionally relevant genomic regions, we compared the level of binding overlap over a range of genomic domains from promoter proximal (within 500 b of a TSS) to more distal regions (Table 3). We found a low though consistent overlap correlation value (∼0.3) whether promoter proximal or distal sites were included in the analysis (Table 3). To confirm that the OCV result is independent of the mean region sizes of the two datasets, we fixed the PR region sizes to 300 bases from each side of peak summits to match mean ER*α* region length (mean = 601) and performed the OCV test again. This did not change the OCV (0.33) for the whole genome dataset, and there was negligible change in OCV observed for other domains (Table 3).

**Table 3:** BiSA Overlap Correlation Value (OCV) testing. BiSA Statistical analysis of overlap between ER*α* and PR datasets using different domain datasets.

Domain	Overlap Correlation Value (OCV)			# of overlaps^b^/total ER*α* regions in domain
	Query = ER*α* Reference = PR	Query = PR Reference = ER*α*	Query = ER*α* Reference = PR (600 bp long)^a^	
Whole Genome	0.33	0.26	0.33	4,344/18,560
500 bp upstream, downstream of TSS	0.3	0.17	0.22	112/419
1 kb upstream, downstream of TSS	0.28	0.18	0.25	157/647
5 kb upstream of TSS	0.3	0.21	0.28	304/1,224
5 kb upstream, downstream of TSS	0.31	0.22	0.3	522/2,147
10 kb upstream, downstream of TSS	0.31	0.22	0.3	929/3,666
45 kb–55 kb upstream of TSS	0.29	0.21	0.28	449/1,929
95 kb–105 kb upstream of TSS	0.31	0.24	0.3	514/2,017
90 kb–110 kb upstream of TSS	0.31	0.23	0.3	878/3,495

**Notes.**

a PR regions are fixed to 600 bp long by cutting off 300 bp on both sides of peak summits.

b Number of overlaps in this column is reported by selecting ER*α* as the query and PR as the reference dataset.

**Figure 5:**
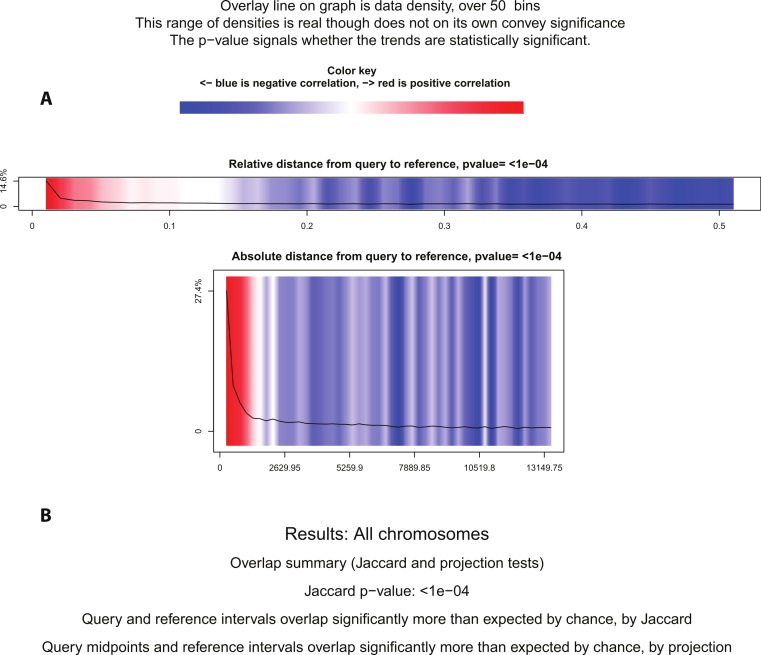
Statistical significance test using Genometricorr. Genometricorr statistical significance analysis of ER*α* (query)-PR (reference). (A) Relative and Absolute Distance Correlation tests are shown graphically. Overlay line (data density) when in the blue section shows negative correlation while the high density in the red section shows positive correlation. (B) Results from Jaccard and Projection tests are shown in text.

Using BITS and Genometricorr, we further investigated whether the spatial proximity correlation between PR and ER*α* binding was more significant than expected by chance. BITS Monte Carlo simulation reported that the spatial correlation of ER*α* and PR was statistically significant, with a *p*-value of 0.0001. Similarly Genometricorr’s Relative Correlation test, Absolute Distance test, Jaccard test and Projection tests also reported the spatial correlation between the two factors as statistically significant (*p*-value =<1e–04) (Fig. 5). We repeated the tests for the 600bp fixed-width PR dataset and found no change in reported *p*-values from BITS or Genometricorr. This confirmed that a change in average region size between the two datasets does not affect the statistical analysis and demonstrated that the tendency for binding events for the two factors to be close to each other is statistically significant. Therefore we conclude that, although there are a number of statistically significant shared binding sites in the ER*α* and PR datasets, and that ER*α* and PR often bind in proximity to each other, the observed overlap of the two factors is not strong enough for them to be considered as co-factors that consistently co-operate on shared binding regions. However, the close proximity of the binding regions for the two factors shows a spatial convergence and is statistically significant.

### Motif analysis

The 4,358 common sections of ER*α*-PR were searched for known motifs. Known motif analysis in these common sections revealed a strong presence of ERE, forkhead protein and PRE motifs. In Table 4, we listed the top ranked motifs, ordered by *p*-value. A PRE motif was found in 41.88% (1,825) of the total 4,358 regions, which was much higher than the number of ERE motifs detected 14.3% (623) of the sequences. However, this may reflect the higher stringency of the position specific scoring matrix used to identify ERE motif occurrence than the matrix used to find PRE motifs since the *p*-value for ERE motif detection (1e–291) was much stronger than the *p*-value for PRE motif occurrence in the dataset (1e–179). The presence of FOXA1 motifs in these regions confirms that the factor facilitates the binding of ER*α* and PR on these regions as previously reported (Augello, Hickey & Knudsen, 2011; Bernardo & Keri, 2012; Nakshatri & Badve, 2009). In addition AP-2 and TEAD4 (TEA) motifs were also identified in these regions and in the 1,000 top scoring PR binding regions. AP-2 has a known role in normal mammary development and breast cancer (Cyr et al., in press; Lal et al., 2013; Woodfield et al., 2010). TEAD4 has also been shown to be co-expressed with other oncogenes and is correlated with poor prognosis (Xia et al., 2014; Mesrouze et al., 2014; Lim et al., 2014). The presence of the related motifs in the ER*α*-PR shared regions as well as in regions that bind uniquely ER*α* or PR suggests that AP-2 and/or TEAD play a key role for both receptors and could be important in facilitating cooperation between the two nuclear receptors.

**Table 4:** Known motif analysis of ER*α* and PR overlapping common regions. Top ranked known motif analysis of ER*α*-PR common sections (4,358 regions).

Motif	Name	*P*-value	% of targets sequences with motif
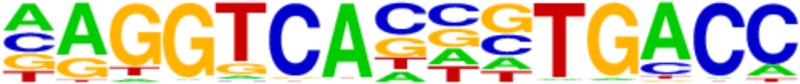	ERE(NR/IR3)/MCF7-ERa	1e–291	14.30%
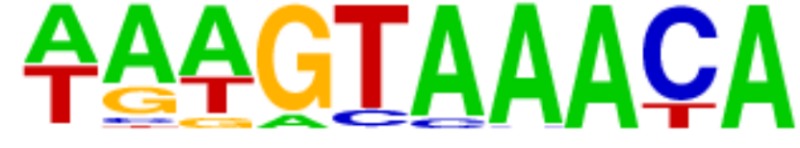	FOXA1(Forkhead)/LNCAP-FOXA1	1e–249	35.11%
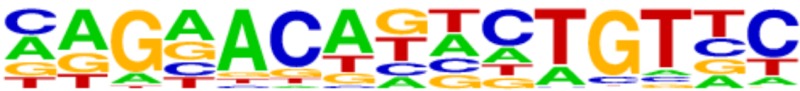	PR(NR)/T47D-PR	1e–179	41.88%
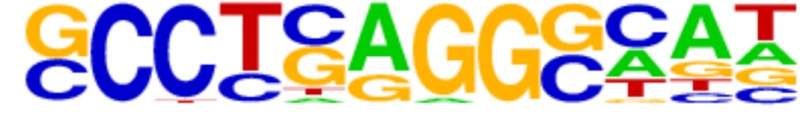	AP-2gamma(AP2)/MCF7-TFAP2C	1e–122	20.38%
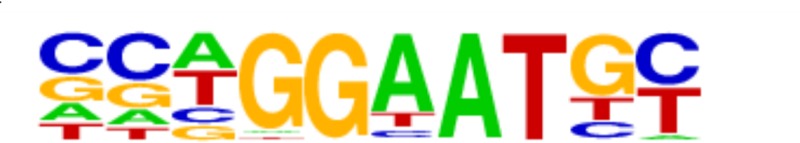	TEAD4(TEA)/Tropoblast-Tead4	1e–86	17.97%

**Figure 6:**
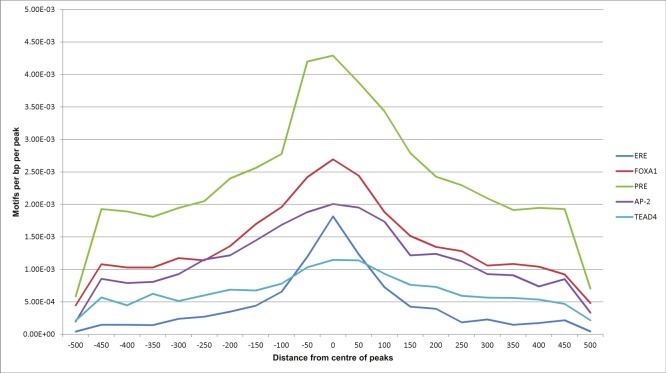
Motif position distributions in ER*α*-PR overlapping regions. Frequency distribution of ERE, FOXA1, PRE, AP-2 and TEAD4 motifs around centres of peaks using a 50 bp bin size.

Using Homer, we also looked at relative position distributions of these motifs (Fig. 6). We found that the motifs converge around the centres of the peaks, supporting their biological significance as primary binding events.

### Enrichment analysis of ER*α*-PR common regions

**Figure 7:**
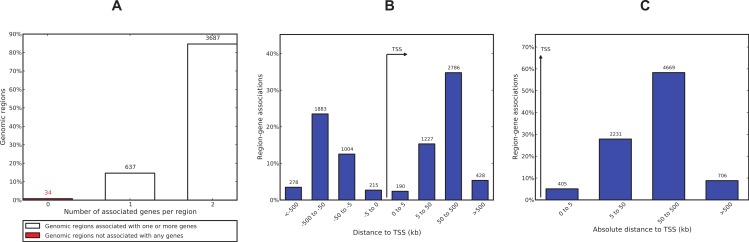
ER*α*-PR common region-gene association. (A) Number of associated genes per region. (B) Region-gene association binned by orientation and distance to TSS. (C) Region-gene association binned by absolute distance to TSS.

We used GREAT (Genomic Regions Enrichment of Annotations Tool) (McLean et al., 2010) to interpret the functional role of 4,358 ER*α*-PR common regions. GREAT revealed that only 34 regions (∼0.8%) are not associated with any gene and 3,687 (∼85%) regions are associated with 2 genes (Fig. 7). Most of the regions were found to be distal binding events while 405 (∼9%) regions are within 5 kb of transcription start sites (TSS). Region to gene association revealed MYC has the maximum number of regions linked to this gene (26 regions). The known role of estrogen-induced MYC oncogene in breast cancer (Orr et al., 2012; Wang et al., 2011a) confirms a biological relevant regions-to-gene association. PGR was also among the top 10 genes identified with the largest number of associated regions (File S1). Gene ontology enrichment analysis of the common regions revealed epithelial cell development as the most significant biological process (File S1). Epithelial cell development was linked to 30 genes associated with 120 regions out of which 4 regions were within 5 kb of a TSS. Pathway Commons, a meta-database of public biological pathway information (Cerami et al., 2006), revealed the ER*α* signalling network as the most significant term (*p*-value = 5.7e–37) where 137 regions were found regulating 24 genes associated with this pathway. The FOXA1 transcription factor network and IL6-dedicated signalling events were also significant terms (*p*-value 1.6e–19 and 2.6e–17). Mouse phenotype analysis revealed two breast cancer related ontologies (abnormal mammary gland epithelium physiology and abnormal mammary gland development) as the most significant terms. There were 32 regions associated with 5 genes linked to abnormal mammary gland epithelium physiology and 189 regions associated with 52 genes linked to mammary gland development. The File S1 also lists regions and associated genes with the ontologies.

## Discussion

The BiSA database provides a good starting point for studying overlapping binding by a range of transcription factors from a comprehensive collection of published studies (Khushi et al., 2014). The datasets available in BiSA represent the original genomic locations identified in the published studies from which they are sourced. Although the same standard pipeline has often been applied, it must be acknowledged that differences in read alignment algorithms (Kerpedjiev et al., 2014; Lunter & Goodson, 2011) and the use of a variety of peak-caller programmes (Ladunga, 2010; Pepke, Wold & Mortazavi, 2009; Wilbanks & Facciotti, 2010) has an impact on downstream analysis, largely due to differences in stringency that affects the number of genomic regions identified. Our initial investigation of the overlap in ER*α* and PR binding in T-47D cells, utilizing the published binding regions, revealed an overlap of ∼27% of ER*α* binding regions with the published PR cistrome (data not shown). This suggested an interesting functional relationship between the receptors, which justified further study. To perform a more rigorous exploration of their overlapping binding patterns, we reanalysed the raw ER*α* and PR ChIP-seq data using a standardized pipeline. This illustrates the great value of BiSA as an easy to implement first pass tool to investigate potential functional relationships in transcription factor binding and epigenomic datasets.

The BiSA statistical overlap correlation value (OCV) represents a statistical summary value of the set of *p*-values calculated by the IntervalStat tool and reflects the overall correlation of two binding site datasets. IntervalStat calculates a *p*-value for each query region against the closest reference region within the given domain. It is designed to identify factors that target the same genomic locations. As described in examples in our previous study (Khushi et al., 2014) the OCV should be greater than 0.5 for partner factors, reflecting a statistically significant correlation between two binding patterns. For example the OCV for known partners, FOXA3 (query) to FOXA1 (reference) was 0.72 (Motallebipour et al., 2009). Similarly the OCV for CTCF (query) and SA1 (reference), which are known to co-locate on DNA, was 0.82 (Schmidt et al., 2010). Therefore the lower OCV for ER*α*-PR suggests that the majority of ER*α* and PR binding events are independent of each other, however, the OCV test does not challenge the biological co-occurrence of binding of the two factors on the reported regions where IntervalStat reports a statistically significant *p*-value.

A consistent overlap was found both proximal and distal to gene promoters (Table 3). It is acknowledged that gene expression is regulated through interaction at a number of cis-regulatory elements, which includes promoters and enhancers. Moreover, enhancers can spread over a range of distances from the TSS. Therefore, the detection of binding sites over a range of distances and locations is to be expected (Bulger & Groudine, 2011; Calo & Wysocka, 2013). This spatial correlation between the two factors is identified as statistically significant by Monte Carlo simulation using BITS, Relevant Distance, Absolute Distance, Jaccard and Projection tests using Genometricorr. Therefore, the regions from the two factors are found in close proximity more often than expected by chance although they do not exactly overlap. Therefore the consistent OCV observed using various domains and statistically significant spatial convergence suggest that the consistent overlap may have biological significance. Although not all sites overlapped, many of the shared ER*α* and PR binding regions were highly statistically significant binding sites for both receptors, as determined by a strong *p*-value and low FDR value in MACS, suggesting that these are biologically valid binding regions for these receptors and that their overlap reflects converging function on a subset of gene targets.

In recent years a number of studies have published ER*α* binding regions in the MCF-7 cell line (Grober et al., 2011; Gu et al., 2010; Hu et al., 2010; Hurtado et al., 2008; Joseph et al., 2010; Schmidt et al., 2010; Tsai et al., 2010; Welboren et al., 2009). However only two studies have published ER*α* data in T47D cells (Gertz et al., 2012; Joseph et al., 2010). We chose to study the Gertz et al. (2012) dataset because using data from the Joseph et al. (2010) study we called only 1,817 peaks with FDR <5%, which can be an indication of low quality ChIP (Landt et al., 2012). On the other hand for the PR dataset, we did not employ the datasets published by Yin et al. (2012) because the experiment was performed with an antiprogestin (RU486) treatment, which would not be expected to elicit the same binding pattern as PR agonist, and lacked any control sample. MACS distributes read tags from the control sample along the genome to model Poisson distribution, and false discovery rate (FDR) is calculated by swapping control and ChIP samples. Therefore it is recommended for ChIP-seq studies to have an appropriate input control sample (Wilbanks & Facciotti, 2010). ENCODE guidelines also emphasise the importance of using a suitable control dataset to adjust for variable DNA fragment lengths (Landt et al., 2012).

There is a slight difference in the reported low-significance motifs for PR data between this report and the Clarke and Graham study (Clarke & Graham, 2012). The two most significant motifs (PRE are FOXA1) are the same in the two studies, however, Clarke and Graham found an NF1 half-site as one of the significant motifs and AP-1 sites as non-significant while in this study we found an AP-2 motif higher in significance than the NF1 motif (not shown). This minor difference is due to the difference in binding regions as Clarke and Graham published 6,312 PR bound regions in T47D cells by aligning to hg18 and using the ERANGE peak caller, however, in this study we reported 22,152 PR regions by aligning to hg19 assembly and using MACS as our peak caller.

The ER*α*-PR data was collected from two separate publications where the binding of each factor was studied by stimulation of T-47D cells with estrogen or progesterone independently. Therefore the focus of this study was to examine the correlation of ER*α*-PR binding patterns which revealed an interesting convergence on specific loci. We studied the association between common regions and nearby genes and found biologically relevant gene pathways. The Myc oncogene, which was most highly associated with binding sites common to ER*α* and PR, is a known target of both estrogen and progesterone and plays a key role in the normal breast and breast cancer (Curtis et al., 2012; Hynes & Stoelzle, 2009). PR itself is also regulated by both hormones and the PGR gene was highly associated with shared ER*α* and PR binding regions. Transcriptional regulation by estrogen and progesterone co-treatment in this cell model was not available, however it would be interesting to study the binding of the two factors under the influence of both stimuli (estrogen and progesterone) to observe the impact of converging ER*α* and PR regulation in comparison to individual stimulation.

## Conclusion

In summary, we have evidence for a biologically relevant interplay between PR and ER*α* in a subset of binding sites in breast cancer cells. Our analysis demonstrated the utility of our previously published software BiSA (Khushi et al., 2014), which has a comprehensive knowledge base, consisting of transcription factor binding sites and histone modifications collected from previously published studies. Using BiSA we identified that ER*α* and PR co-locate on a subset of binding sites. The BiSA statistical testing of overlap revealed a low overlap correlation value (OCV) suggesting that the two factors are not obligate cofactors. However, spatial correlation testing using Monte Carlo simulation by BITS, Relevant Distance, Absolute Distance, Jaccard and Projection tests by Genometricorr revealed a statistically significant correlation between the two factors. In addition, the discovery that ER*α*, FOXA1, PR, AP-2 and TEAD4 binding motifs are significantly enriched in regions that are bound by both ER*α* and PR suggests that their overlap is biologically relevant.

## Supplemental Information

10.7717/peerj.654/supp-1 File S1 Enrichment analysis of ER*α*-PR common regions using GREAT
